# Screening for depression as part of annual diabetic review using PHQ-9 scores: a pilot study

**DOI:** 10.1017/S1463423623000294

**Published:** 2023-06-20

**Authors:** Rabeeah Asim, Muhammad Asim, Rajah Reddy, Lynne Chepulis, Ross Lawrenson

**Affiliations:** 1 Medical Assistant at Tui Medical, Rototuna at Time of Data Collection, Taranaki Base Hospital, New Plymouth, New Zealand; 2 General Practitioner, GP at Tui Medical, Rototuna at Time of Data Collection, TuiOra Family Health, New Plymouth, New Zealand; 3 Waikato District Health Board, Hamilton, New Zealand; 4 Waikato Medical Research Centre, Te Huataki Waiora School of Health, University of Waikato, Hamilton, New Zealand

**Keywords:** depression, diabetic annual review, PHQ-9 score, type 2 diabetes mellitus

## Abstract

Diabetes mellitus is associated with an increased risk of depression. Appropriate screening and treatment of depression may therefore support diabetes management in primary care. Study aim was to review the efficacy of using a Patient Health Questionnaire – (PHQ-9) tool to screen for depression in patients with type 2 diabetes mellitus (T2DM) in New Zealand.

It was a cross-sectional study that included 100 consecutive patients with T2DM from two urban practices in Hamilton, New Zealand. Patients were screened using PHQ-9 scores.

Using the PHQ-9, the overall prevalence of depression was 29% including 11 patients under active management/prescribing for depression and 18 undiagnosed patients. By ethnicity, depression affected 41.3%, 33.0%, 25.0% and 13.3% of NZ European, Māori, Pacific and other ethnicities, respectively. PHQ-9 scoring is an easy to administer tool that can be used to screen for unrecognized depression in patients with diabetes as a part of an annual diabetic review.

## Background

Diabetes is a heterogeneous set of disorders characterized by glucoregulatory abnormalities. Worldwide prevalence of diabetes is 9.8% in men and 9.3% in women (Danaei *et al.*, 2011). In New Zealand, the overall prevalence of diabetes is approximately 7% and this is higher in men than women (8.3% vs 5.8%), and in Māori (7.4%) as compared to NZ Europeans (5%) (Coppell *et al.*, 2013). Importantly, type 2 diabetes mellitus (T2DM) is associated with an increased risk of incident and recurrent depression, with evidence suggesting a bidirectional association between the two (Pan *et al.*, 2010). In New Zealand, up to one quarter of patients with T2DM are currently taking an antidepressant medication, with use more likely in women, New Zealand European, obese patients and those receiving multiple medications. (Chepulis *et al.*, 2020).

While medication use for depression is relatively uncommon in T2DM, less is known about the prevalence of the condition. International data suggested that at least half of people with T2DM suffer with depression (Sartorius, 2022), and New Zealand reports on the strong association between mental health and chronic disease (Scott *et al.*, 2006). However, it has been suggested that a large proportion of depression goes undiagnosed in this population, either due to clinical inertia or a range of patient factors (Sartorius, 2022).

The importance of managing depression in diabetes patients should not be understated, though comorbidity of T2DM and depression can interfere with and limit the effectiveness of treatment for diabetes. Antidepressant medications may have a direct effect on glycemic control that are independent of its effect on weight and mood (Khapre *et al.*, 2020), and consequences of depression in diabetic patients can be chronic and severe: the presence of depression in a person with diabetes may lead to 36.8% increase in coronary artery disease and a 47.9% increase in cardiovascular mortality (Farooqi *et al.*, 2019).

An integrated approach involving treatment of both the mental health condition and diabetes has been shown to be the best approach for improving outcomes in primary care, including improved HBA1c levels and remission of depression symptoms (Sartorius, 2022). However, for clinicians to be able to appropriately manage all relevant conditions, they must be first identified. Thus, this small study aims to explore the efficacy of using a Patient Health Questionnaire – 9 (PHQ-9) tool for screening for depression in people with diabetes.

## Methods

For this pilot study, a cross-sectional study method was used: 100 consecutive patients with T2DM who presented for repeat medications and/or annual diabetic review at one of two specific medical clinics in Hamilton were screened for depression using the PHQ-9, a valid and reliable screening tool for detecting depression (Atlantis *et al.*, 2017). The population at these clinics is a mix of high need patients including pacific and Māori (total registered patients around 13 000). Patients were included if they were aged 18–85 years and were at least 1-year post-T2DM diagnosis.

Data were collected from April to October 2022, either by face to face or phone consultations (during COVID lockdowns). Other data including BMI (height and weight), age, sex, ethnicity, HBA1c level, current use of antidepressant, current diabetes and cardiovascular medications and duration of diabetes were collected from the practice electronic patient management system. The data were collected by the primary author, a general practitioner at one of these clinics and his trainee intern. Depression was scored as mild (5–9), moderate (10–14) or severe (above 14) (Manea *et al.*, 2015). This study got Ethics Approval from Health and Disability Ethics Committee (HDEC), New Zealand with approval number RM8309 https://ethics.health.govt.nz/.

## Results

The demographics of the study population is given in Table 1. The mean age of patients was 59 years (range 18–85 years), and 59% of participants were male. Twenty nine percent of patients were New Zealand European, 9% were Māori with the remainder being of Pacific (16%), Asian (15%) and Other (31%) descent. The mean time since diabetes diagnosis was 10 years (Table 1).

**Table 1.** Study population demographics

Age	18–85 (mean 59 ± 14.2)
Sex	59 males and 41 females
Ethnicity	European 30%, Indian 23%, Pacific 16%, Asians 14%, Māori 9% and Africans 8%
HBA1c	Mean 60.6 ± 17 (range 29–118)
CVD score	13.5 ± 6.8 (range 1–39)
BMI	Mean 31.5 ± 8.3 (range 19–58)
BP – systolic	130 ± 14.6 (range 95–180)
BP – diastolic	77.5 ± 8.8 (range 60–100)
Year of diagnosis	1973–2020 (1–48 years) mean 10 years

Using the PHQ-9 tool, 18 patients were found to have a score of ≥ 5, of which two were currently prescribed a selective serotonin reception inhibitor (SSRI). A further 11 patients had a score of <5 but were active users of antidepressant medication (six were using SSRIs, four were prescribed other antidepressants such as venlafaxine and one patient was prescribed benzodiazepines). Overall, the prevalence of depression (known and previously unknown) was 29/100 (29%). Of the 18 previously undiagnosed patients, 8 had moderate to severe depression (requiring urgent active clinical management of depression) and 10 had mild depression.

By ethnicity, total and previously undetected prevalence was 41.3%/24.1% for European patients, 33.0%/33.0% for Māori patients, 25.0%/25.0% for Pacific patients, 13.3%/6.7% for Asian and 21.7%/6.5% of those of “Other” ethnicities.

Overall, 67 out of 100 patients were prescribed angiotensin-converting enzyme inhibitors/angiotensin receptor blockers with 25 patients regularly prescribed other forms of anti-hypertensive medications. Similarly, 68% patients were prescribed statin medications.

In our small cohort, there were no obvious differences between the demographics of those T2DM patients with and without depression with regard to age, sex, HBA1c, Cardio Vascular Disease (CVD) risks, blood pressure or duration of diabetes (Table 2).

**Table 2.** T2DM patient descriptors for those with and without depression

	T2DM with depression	T2DM with no depression
Age	61.5	58.8
Sex	15M, 13F	44M, 28F
HBA1c	63	60
BMI	33	30
CVD risk, %	14	13
BP (mean)	130/79	129/77
Duration of T2DM (mean years)	10 years	10 years

## Discussion

Previously, undetected depression was common in our small cohort of T2DM patients, and the PHQ-9 tool was shown to be an easy-to-administer and effective screening tool in this group. Although our pilot study group was not collected in a way to be representative of the local demographic or the general practice population, it did identify that a large proportion of Māori, European and other patients were potentially being missed for treatment or intervention of depression. This aligns with other research that suggests that depression is often under-diagnosed or under-treated in diabetes (Sartorius, 2022), including in New Zealand Māori (Atlantis *et al.*, 2017; McClintock *et al.*, 2022).

While our study is small, it does indicate that depression affects up to 30%–40% of patients with T2DM, which is comparable to that reported elsewhere (Egede & Ellis, 2010; Alhunayni *et al.*, 2020). We suggest that while depression is recommended to be a routine part of diabetes screening (The Annual Diabetes Review, 2022), it should be fully integrated into the annual diabetic review including the portal forms. We also suggest that it should be routinely used for opportunistic screening tool for patients coming for repeat prescriptions/follow-up, noting that effective communication between health professionals and patients is required to tease out additional concerns during these routine visits (Dowell *et al.*, 2018).

This was a small cross-sectional study localized to only two clinics in Hamilton which may not represent the wider Waikato region or diverse New Zealand population. Our data do suggest, however, that a larger, multicenter study is required to explore the prevalence of undiagnosed depression in patients with T2DM, particularly in those from different ethnic groups. Future studies should also include a comprehensive review of patients with mild depression who may be being managed without medication as this was not evaluated in our cohort. Further, it would be interesting to correlate these data to medication compliance and prescribing data, as Māori and Pacific people often seek help late and/or are under-prescribed mental health medications (Farooqi *et al.*, 2019).

Lastly, we note that depression is rarely seen in primary care in isolation and it often presents alongside anxiety and other conditions. Thus, while the PHQ-9 tool has been demonstrated to be effective at identifying patients with undetected depression, it may be more useful to use this alongside other tools such as the GAD-7 for anxiety and or the generic Kessler-10 (Vasiliadis *et al.*, 2015). These tools should be evaluated in further studies.

## Conclusion

PHQ-9 scoring is an easy-to-administer tool to screen for depression in patients with diabetes, with data suggesting that approximately a quarter of patients may be undiagnosed. We suggest that PHQ-9 screening should be an integral part of the annual diabetic review for early detection and management of depression in patients with diabetes.

A limitation of this pilot study is that this study design may have missed patients with mild depression that was being managed without medication; however, these should be included in future studies.
